# Integrating addiction medicine training into medical school and residency curricula

**DOI:** 10.1186/1940-0640-10-S1-A28

**Published:** 2015-02-20

**Authors:** Jan Klimas, Launette Rieb, Gerard Bury, John Muench, Thomas O’Toole, Traci Rieckman, Walter Cullen

**Affiliations:** 1 Department of Family Practice, Faculty of Medicine, University of British Columbia, Vancouver, BC, Canada, V6Z 1Y6; 2 School of Medicine and Medical Science, University College Dublin, Coombe Family Practice, Dublin 8, Ireland; 3 Department of Family Medicine, Oregon Health & Science University, Portland, OR, 97239-3098, USA; 4 Department of Medicine, Providence VA Medical Center, Providence, RI, 02908, USA; 5 Department of Public Health and Preventive Medicine, Oregon Health & Science University, Portland, OR, 97239-3098, USA; 6 Graduate Entry Medical School, University of Limerick, Limerick, Ireland

## Background

The Affordable Care Act (2010) brings an opportunity to increase the integration of addiction treatment into the health-care system. With the anticipated expansion of addiction care services in primary care, challenges, such as workforce training, can be expected. This presentation discusses challenges and opportunities for addiction medicine training of primary care professionals in Ireland, Canada, and Portland, Oregon.

## Objectives

To explore ideas for integrating addiction medicine education into medical school, fellowship, and residency curricula and to consider how implementation barriers can be addressed.

## Method

The presentation will outline the setup and content of some of the current addiction medicine education in medical schools and residency programs in Ireland, Canada, and Portland, Oregon. Examples from three educational initiatives will be used to generate ideas applicable to each setting and help overcome integration barriers: the St. Paul’s Hospital Goldcorp Addiction Medicine Fellowship (http://www.addictionmedicinefellowship.org), the feasibility study on alcohol SBIRT for opioid agonist patients in Ireland (PINTA), and the team-based SBIRT Oregon project (http://www.sbirtoregon.org). Scenarios that illustrate implementation strategies, such as educational outreach visits to practitioners—based on overcoming obstacles to change—and facilitators of integrating addiction medicine education into medical school and residency curricula, will be described.

## Conclusion

The presentation will conclude with an overview of how initiatives in which the authors have been involved may be used to improve addiction medicine education.

